# Supplementary material for: Identification and functional analysis of non-coding regulatory small RNA FenSr3 in Bacillus amyloliquefaciens LPB-18
Source: PeerJ. 2023 May 15;11:e15236. doi: 10.7717/peerj.15236 (PMC10194069; doi:10.7717/peerj.15236)
Supplement: Supplemental Information 4 [file peerj-11-15236-s004.zip › KO/CK-vs-T1_map/map05140.html]

KEGG PATHWAY: Leishmaniasis - Reference pathway


|  |  |
| --- | --- |
| **Leishmaniasis - Reference pathway** |  |

[
Pathway menu
| Organism menu
| Pathway entry
| Show description
| User data mapping
]

Leishmania is an intracellular protozoan parasite of macrophages that causes visceral, mucosal, and cutaneous diseases. The parasite is transmitted to humans by sandflies, where they survive and proliferate intracellularly by deactivating the macrophage. Successful infection of Leishmania is achieved by alteration of signaling events in the host cell, leading to enhanced production of the autoinhibitory molecules like TGF-beta and decreased induction of cytokines such as IL12 for protective immunity. Nitric oxide production is also inhibited. In addition, defective expression of major histocompatibility complex (MHC) genes silences subsequent T cell activation mediated by macrophages, resulting in abnormal immune responses.

|  |  |  |
| --- | --- | --- |
| Reference pathway | 184% 150% 122% 100% 82% 67% 55% | 图片下载 |
